# Maternal Music Exposure during Pregnancy Influences Neonatal Behaviour: An Open-Label Randomized Controlled Trial

**DOI:** 10.1155/2012/901812

**Published:** 2012-02-14

**Authors:** Ravindra Arya, Maya Chansoria, Ramesh Konanki, Dileep K. Tiwari

**Affiliations:** ^1^Division of Neurology, Cincinnati Children's Hospital Medical Centre, MLC 2015, 3333 Burnet Avenue, Cincinnati, OH 45229, USA; ^2^Department of Pediatrics, N.S.C.B. Medical College, Jabalpur 482003, India; ^3^Division of Pediatric Neurology, Department of Pediatrics, All India Institute of Medical Sciences, New Delhi 110029, India; ^4^Department of Pediatrics, Deen Dayal Upadhyay Hospital, New Delhi 110064, India

## Abstract

*Objective*. This study evaluated the effect of antenatal music exposure to primigravida healthy mothers on the behaviour of their term appropriate-for-date newborns assessed using Brazelton Neonatal Behavioral Assessment Scale (BNBAS). *Methods*. This was a single-centre, randomized, open-label controlled trial. Primigravida mothers aged 19–29 years, free of chronic medical diseases or significant deafness, with singleton pregnancy, with a gestation of 20 weeks or less, were randomized to listen to a pre-recorded music cassette for approximately 1 hour/day in addition to standard antenatal care (intervention arm) or standard care only (control arm). Perinatal factors with adverse effect on neonatal behaviour were deemed as protocol violations. Outcome measure included scores on 7 clusters of BNBAS. Primary analysis was per protocol. The trial is registered with ClinicalTrials.gov (NCT01278329). *Results*. One hundred and twenty-six newborns in the music group and 134 in the control group were subjected to BNBAS assessment. The infants of mothers exposed to music during pregnancy performed significantly better on 5 of the 7 BNBAS clusters. The maximal beneficial effect was seen with respect to orientation (ES 1.13, 95% CI 0.82–1.44, *P* < 0.0001) and habituation (ES 1.05, 95% CI 0.53–1.57, *P* = 0.0001). *Conclusion*. Prenatal music exposure to mother significantly and favourably influences neonatal behaviour.

## 1. Introduction

The developing nervous system in utero is exposed to myriad influences with potentially far reaching consequences. Most of the research in this area is directed towards understanding the adverse influences and their structural or functional pathogenesis [1]. However, it is also attractive to investigate if foetal neurodevelopment can be positively influenced or enhanced in an analogous manner. There is evidence that appropriate vibroacoustic stimulation by exposure to music alters foetal behaviour and is carried forward to the newborn period [2, 3]. Music is a noninvasive, culturally acceptable intervention with multiple putative direct and indirect beneficial effects on mother and foetus through the pregnancy and perinatal period. In animals, prenatal music exposure has been shown to improve postnatal spatial learning and memory; to reduce isolation stress [4]. Music has been found to beneficially affect stress response and recovery from critical illness or surgery [5, 6]. Using optical topography and salivary cortisol as a marker of stress, music has been documented to simulate pleasure and happiness [7]. On a molecular level, music has been shown to alter dominergic neurotransmission and have direct effect on neurotrophic growth factors including brain derived neurotrophic factor and tyrosine kinase receptor B [5, 8]. Besides direct influence on emotions, behavior, and neurotransmitter systems, there are multiple endocrine effects of music exposure including altered levels of adrenal and gonadal steroids. These changes in a pregnant woman can influence neuroblast proliferation, axonogenesis, synaptogenesis, and neuronal organization with effects on cognitive performance and behavioural gestalt. The present study was carried out to test the hypothesis that music exposure to mother during pregnancy can affect the neonatal behaviour.

## 2. Methods

This was a single-centre, open-label, randomized controlled trial (RCT) conducted at a teaching hospital from January 2003 to December 2005. The study was approved by institutional ethics committee and is registered with ClinicalTrials.gov (NCT01278329).

### 2.1. Participants

**Figure 1:**
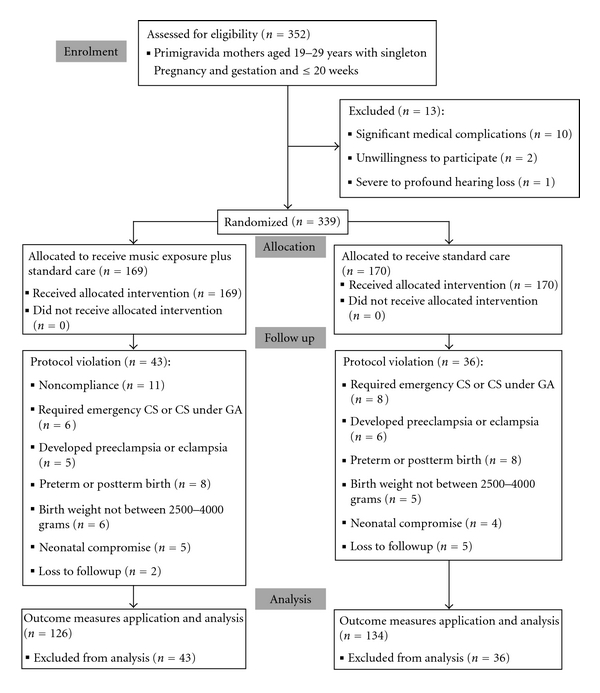


All consecutive primigravida mothers of 19 to 29 years of age with singleton pregnancy attending the antenatal clinic of the study institution first time, at or before 20 weeks of gestation, were eligible for inclusion. Mothers with significant coexisting medical diseases or severe to profound hearing loss were excluded (Figure 1). Demographic details were recorded on a structured data sheet. Mothers were then randomized to music and control groups using a printed random number table. Two groups were generated using block randomization method, using variable length of blocks. Allocation to the groups was concealed from the investigator (RA) performing outcome assessment. All mothers received standard antenatal care. Mothers randomized to music group were given a cassette player and a prerecorded music audio cassette and were demonstrated their use.

### 2.2. Interventions

Mothers in the music group were provided a prerecorded “Garbh Sanskar” audio cassette (Times Music Inc., Mumbai, India) with a running duration of approximately 50 minutes and a cassette player with headphones. This contains a medley of instrumental music, natural sounds, and chants from religious scriptures. They were asked to listen to the recorded music daily in the evening just before going to the bed with a minimum of ambient noise. They were also asked to maintain a record of their music listening activity by making a check mark on a printed calendar. Mothers were then followed up with conventionally scheduled antenatal visits. At each visit, the compliance was ascertained by reviewing the calendar. Criteria for protocol violation included noncompliance with music listening for more than 2 weeks, development of preeclampsia or eclampsia in the mother, delivery of the newborn at a gestation of less than 37 or more than 42 completed weeks, delivery of the baby by emergency caesarean section, requirement of general anaesthesia even in case of elective caesarean section, neonatal birth weight less than 2500 grams or more than 4000 grams, or presence of significant neonatal disease precluding application of outcome assessment. Each protocol violation was counted only once in a mutually exclusive fashion.

### 2.3. Outcomes

All healthy term appropriate for date neonates born of spontaneous vaginal delivery or elective caesarean section conducted under epidural anaesthesia were subjected to outcome assessment. Hence, the primary analysis was per protocol.

Outcome measures consisted of the performance on Brazelton Neonatal Behavioral Assessment Scale (BNBAS). The BNBAS is a means of scoring interactive behaviour for term and stable preterm infants. The scale consists of 27 behavioural items, each scored on a 9-point scale, and 20 elicited responses, each scored on a 3-point scale. In most cases, the infant's score is based on the best performance, not an average performance [9].

The BNBAS was administered once to each infant in the study on day 2 or 3 of life. The assessment was performed by the investigator (R.A.) who has received prior training in its application, and the items were scored as recommended in the manual [9]. Infants were tested midway between feeds in a quiet, dimly lit room with an ambient temperature of 32–34°C. The items were grouped as recommended by Lester into the following 7 clusters: habituation, orientation, motor performance, range of state, regulation of state, autonomic stability, and reflexes [10].

### 2.4. Sample Size Estimation and Statistical Analysis

Sample size estimation for this study presented many challenges. A prior prospective study with similar design used a sample size of 20 believing it to capture “significant differences in fetal behavior” [3]. A pilot study was not feasible because of long follow-up period from enrolment of the mother to delivery of the newborn; lack of single primary outcome measure. Hence, it was decided to conduct the study in an open-ended manner limited by time of enrolment (January 2003 to March 2005) rather than number of mothers enrolled.

The data was entered in a Microsoft Excel spreadsheet (MS Office version 2003). Mean scores in each cluster were compared using *t*-test for independent samples. Effect size and 95% confidence intervals (CI) for the same were calculated. Baseline variables were compared using *t*- and *χ* ^2^-statistics. The open source freeware “OpenStat” was used for calculations [11]. All mothers gave written informed consent before enrolment. The study was approved by institutional ethics committee.

## 3. Results

A total of 352 primigravida females attending antenatal clinic for the first time at a gestation of 20 weeks or less were evaluated for participation. Ten females were excluded because of chronic medical diseases including rheumatic heart disease [5], chronic hepatitis [2], uncontrolled type 1 diabetes, chronic obstructive pulmonary disease, and vesicoureteric reflux-associated chronic renal failure (1 each). Two females were unwilling to participate, and 1 was found to have 90 dB hearing loss on audiometric evaluation. The remaining 339 females were randomized to receive music exposure in addition to standard antenatal care (intervention arm, *n* = 169) and standard care alone (control arm, *n* = 170). The groups were comparable at baseline (Table 1). The primary analysis was per protocol, and BNBAS assessment was applied to 126 newborns in the music exposure group and 134 newborns in the control group (Figure 1).

The infants born to mothers exposed to music during their pregnancy scored significantly higher on 5 of the 7 BNBAS clusters including habituation, orientation, range of state, regulation of state, and autonomic stability. In all these clusters, the 95% confidence interval (CI) for effect size (ES) remained on 1 side of the point of no difference (Table 2). The maximal beneficial effect was seen in the clusters of orientation (ES 1.13, 95% CI 0.82–1.44, *P* < 0.0001) and habituation (ES 1.05, 95% CI 0.53–1.57, *P* = 0.0001). The newborns of music exposure group also showed a significant trend towards better motor performance (ES 0.25, 95% CI 0.0–0.5, *P* = 0.0479); however, the lower bound 95% CI touched the point of no difference. There was no difference between the infants of intervention and control arms on the reflexes cluster.

There were 43 (25.4%) protocol violations in the mothers randomized to music group and 36 (21.2%) in the control group (*z* = 0.9292, *P* = 0.3528). The breakup of causes for protocol violations is provided in Figure 1. Compliance for listening to music was assessed using self-maintained record. The mean duration of music exposure in mothers of intervention arm was found to be 173.3 (±18.9) hours.

## 4. Discussion

The present study supports the hypothesis that maternal exposure to music during pregnancy can beneficially influence neonatal behaviour. Behavioral responses test the integrity of neonatal nervous system at several levels including perception, afferent conduction, integration, conscious decision, and efferent motor apparatus [9].

The maximum effect of music exposure was seen in the orientation cluster (mean difference 1.13 points) (Table 2). Orientation items test the infants' response to animate and inanimate, auditory, and visual stimuli presented separately or together and constitute the “Social Interactive package” of BNBAS [9]. The mean score of infants belonging to music group in this cluster was 6.5 which implies that the average infant was able to follow the visual stimulus with smooth coordinated movement of head and eyes in 30–60° arcs horizontally and probably also vertically; exhibited alerting and searching behaviour in response to sound stimulus [9].

The habituation cluster also showed significantly better scores in infants born to mothers exposed to music during pregnancy (mean difference 1.05 points) (Table 2). The “Habituation package” of BNBAS tests response decrement to repeated stimuli, including visual (light), auditory (rattle and bell), and tactile (pin prick to foot) stimuli [9]. The average infant in the intervention arm scored 5.7 in this cluster which implies shutdown of body movements and some diminution of blinks and respiratory changes after few repetitions of visual or auditory stimuli. For the tactile stimulation item, this score implies a response localized to stimulated leg or foot after 5 trials with no movement in rest of the body [9]. Such motor behaviour belongs to the Volpe's category of “high level” responses which depend on intact integration function in central nervous system (CNS) [12].

The infants of the mothers of music group also showed significantly better performance than the control group with respect of range and regulation of behavioural states and autonomic stability (Table 2). The neonatal infant displays a rich repertoire of behavioural states; the interplay of these states, their transition, and variety presented by the newborn is akin to examining the “higher mental functions” of the adult. There was also a trend towards better motor performance in the infants belonging to intervention arm, but it failed to reach statistical significance.

The effects of maternal experiences on foetal or neonatal behaviour have been studied previously and explored for the possibility of modifying this behaviour. A prospective RCT studied the effect of music played to 10 foetuses (median gestation 38 weeks) with a headphone on the maternal abdomen. A silent headphone taped to abdomen of another 10 mothers comprised the control arm. The exposed foetuses showed higher mean heart rates (FHR) and higher FHR variation in the first hour itself, with significantly more state transitions by fourth hour. These newborns also showed more state transitions and spent a higher proportion of time in awake state, when exposed to same music stimulus after birth [3]. The authors concluded that this suggests the occurrence of a simple form of foetal programming or learning.

Another study has been conducted to examine whether foetal response to music differs from that to human voice. Ten healthy term foetuses were exposed to music, voice, and sham in random order for three 15 second intervals. Foetuses were found to respond by increased FHR and motor response to both music and voice which was significantly different from sham exposure but not different between themselves [2]. It has also been demonstrated that foetal repertoire of responses to music exhibits a pattern of maturation with the gestation. In response to piano recordings, younger foetuses (28–32 weeks gestation) responded by transient increase or decrease in heart rate depending on sonic intensity, probably indicating selective attention to stimulus; whereas the more mature foetuses displayed sustained elevation in heart rate (>33 weeks) and change in body movements (35 weeks) [13]. The authors concluded that processing of complex sounds changes at 33 weeks of gestation [13].

 Experience in the present study agrees with published literature that music exposure in utero does influence neonatal behaviour. However, there are certain important differences. This study enrolled mothers in first half of pregnancy and the foetus was exposed to a mean duration of 173 hours of music before birth, whereas other studies have exposed the foetus only for a few hours prior to birth. Also, the present study used conventional headphones worn by the mother over her ears instead of the one taped to her abdomen as in other studies. Although this would likely have resulted in less direct sonic stimulation of the foetus, the practical implications of this approach are more because of its better adaptability to routine clinical practice. A limitation of the present study was no standardization of the intensity of music stimulus. However, this might be relevant in case of directly applied stimulus over maternal abdomen where it conveys both vibratory and acoustic sensations [14], but not in present circumstances where it was better to let individual mothers decide about the volume of music as per their convenience.

The onset of foetal hearing occurs at about 24 weeks of gestation [15]. In the present study, mothers were exposed to music from early gestation (≤20 weeks). It is not known when the favourable effect of maternal music exposure started, and hence optimal timing for such stimulation in clinical practice cannot be ascertained. It is improbable in the present study that music directly had any auditory effects on the foetus. The effects are more likely to be mediated via endocrine changes produced in the mother. Music is known to have multiple endocrine effects including increased growth hormone which modulates the production of certain cytokines, increased ovarian steroid secretion, changes in the biorhythms and levels of cortisol, testosterone, and estrogen [5, 16]. Corticosteroids have several regulatory effects on growth of neuroblasts, myelination, and metabolism in developing brain [17]. They have been demonstrated to influence important enzymes, for example, sodium-potassium ATPase, and growth factors, for example, basic fibroblast growth factor (bFGF-2) in developing cerebrum in animals [17]. Over 200 steroid responsive genes have been identified in the rat hippocampus involved in axonogenesis, synaptogenesis, cell adhesion, and signal transduction [17]. Thus, music exposure in the mother might influence neurogenesis and cerebral plasticity in the foetus through mechanisms mediated by steroids.

In conclusion, this study provides preliminary evidence that maternal music exposure beneficially affects neonatal behaviour. A trained clinician can utilize the behavioural organization of the newborn infant to gain insights into the intrauterine experience and the perinatal events which may have influenced the neonate's CNS organization [9]. The present clinical trial was not designed to study these aspects and provides no information regarding the mechanism behind the observed effect. Further studies should confirm this observation with a more rigorous design and try to elucidate the direct and endocrine-mediated mechanisms of the effect of music on foetus and newborn.

## Figures and Tables

**Table 1:** Baseline comparisons of relevant maternal and neonatal variables between music and control groups.

Variable	Music group (*n* = 126)	Control group (*n* = 134)	Test statistic	*P* value
*Maternal factors*				
Maternal age (years) (mean ± SD)	23.8 ± 1.9	24.9 ± 2.3	*t* = −1.14, *df* = 258	0.2542
Gestational age at enrolment (completed weeks) (mean ± SD)	13.1 ± 2.4	12.7 ± 2.9	*t* = 1.21, *df* = 258	0.2283
Family socioeconomic class (I, II and III, IV, and V)	4, 88, 34	7, 91, 36	*χ* ^2^ = 0.68, *df* = 2	0.7117
Maternal professional status (working, housewife)	45, 81	49, 85	*χ* ^2^ = 0.02, *df* = 1	0.8862
Mode of delivery (vaginal, caesarean section)	97, 29	98, 36	*χ* ^2^ = 0.51, *df* = 1	0.4737

*Neonatal factors*				
Sex (male : female)	69, 57	71, 63	*χ* ^2^ = 0.08, *df* = 1	0.7739
Birth weight (grams) (mean ± SD)	2693.5 ± 94.7	2686.1 ± 89.9	*t* = 0.646, *df* = 258	0.5186

SD: standard deviation; Socioeconomic classes as per Kuppuswamy's socioeconomic status scale 2003 modification: I: upper, II: upper middle, III: lower middle, IV: upper lower, V: lower.

**Table 2:** Comparison of BNBAS clusters scores between music and control groups.

Cluster	Music group (*n* = 126) (Mean ± SD)	Control group (*n* = 134) (Mean ± SD)	Effect size (95% CI)	Test statistic	*P* value
Habituation	5.72 ± 1.9	4.67 ± 2.3	1.05 (0.53, 1.57)	*t* = 3.999, *df* = 258	0.0001
Orientation	6.51 ± 1.1	5.38 ± 1.4	1.13 (0.82, 1.44)	*t* = 7.207, *df* = 258	<0.0001
Motor performance	4.56 ± 1.2	4.31 ± 0.8	0.25 (0.00, 0.50)	*t* = 1.987, *df* = 258	0.0479
Range of state	4.35 ± 0.5	4.04 ± 0.6	0.31 (0.17, 0.45)	*t* = 4.511, *df* = 258	<0.0001
Regulation of state	4.33 ± 1.0	3.79 ± 1.1	0.54 (0.28, 0.80)	*t* = 4.134, *df* = 258	<0.0001
Autonomic stability	5.88 ± 0.7	5.62 ± 0.9	0.26 (0.06, 0.46)	*t* = 2.589, *df* = 258	0.0102
Reflexes	5.19 ± 1.9	5.24 ± 2.4	−0.05 (−0.58, 0.48)	*t* = −0.185, *df* = 258	0.8530

SD: standard deviation, CI: confidence interval.
